# *C. albicans* Detection with Electrochemical Sensors by Using Molecular Imprinted Polymer Technique

**DOI:** 10.3390/polym18060770

**Published:** 2026-03-22

**Authors:** Naphatsawan Vongmanee, Jindapa Nampeng, Chuchart Pintavirooj, Sarinporn Visitsattapongse

**Affiliations:** Department of Biomedical Engineering, School of Engineering, King Mongkut’s Institute of Technology Ladkrabang, Bangkok 10520, Thailand; naphatsawan.v@hotmail.com (N.V.); jindapa.na@kmitl.ac.th (J.N.); chuchart.pi@kmitl.ac.th (C.P.)

**Keywords:** *Candida albicans*, molecular imprinted polymer technique, screen printed electrode

## Abstract

*Candida albicans* (*C. albicans*) is an opportunistic fungal pathogen and a major cause of nosocomial infections, especially in immunocompromised patients. Conventional diagnostic approaches such as blood culture and biochemical assays are accurate but require multi-step sample processing and prolonged turnaround times, limiting their applicability for rapid clinical screening. In the present study, we developed an electrochemical biosensor based on molecularly imprinted polymer (MIP) technology for the rapid and selective detection of intact *C. albicans* cells. The MIP layer was electropolymerized onto a screen-printed carbon electrode (SPCE), forming selective recognition cavities complementary to the fungal morphology. Electrochemical characterization and detection were performed using cyclic voltammetry in phosphate-buffered saline (PBS). The system demonstrated a wide linear detection range, enabling reliable quantification of *C. albicans* across concentrations spanning from 1 to 10^4^ CFU/mL and achieved an ultralow limit of detection (LOD) of 1.30 CFU/mL, demonstrating high sensitivity. High selectivity was confirmed against *E. coli*, *S. aureus*, and *P. aeruginosa*, demonstrating that the imprinted cavities effectively distinguish fungal cells from bacterial contaminants. These findings highlight the promise of MIP-based electrochemical biosensors as a simple, low-cost, and portable alternative for early fungal diagnostics.

## 1. Introduction

*C. albicans* is a yeast-like fungus that commonly inhabits the human body, particularly the oral cavity, skin, and gastrointestinal tract [1,2]. In healthy individuals, it remains in small quantities without causing harm. However, when the natural microbial balance is disrupted, *C. albicans* can overgrow and lead to infections collectively known as candidiasis [3,4]. This organism is frequently associated with vaginal yeast infections [5,6], diaper rash [7], and oral thrush [8]. In more severe conditions, the imbalance between *C. albicans* and protective bacteria may result in invasive candidiasis, a systemic infection that can spread through the bloodstream and affect major organs including the heart, blood, bones, and brain [9,10]. Morphologically, *C. albicans* typically appears in a yeast form, consisting of round to oval white cells with a diameter of approximately 5–8 μm [11,12].

In recent years, nosocomial fungal infections have shown a significant increase, with *C. albicans* recognized as the most prevalent fungal pathogen in hospitalized patients [13,14,15]. This organism is particularly associated with high morbidity and mortality among critically ill individuals [16]. Infections caused by *C. albicans* affect not only the bloodstream but also the skin and mucosal surfaces, leading to a broad spectrum of clinical conditions [17]. Common forms of candidiasis include cutaneous candidiasis, oral candidiasis [18], vaginal candidiasis [18], candida granuloma [19], and invasive candidiasis [20]. Once the fungus gains access to the bloodstream, it can disseminate to internal organs, resulting in life-threatening complications and severe organ-specific infections [21]. Moreover, immunocompromised patients or those exposed to physiological stress are at heightened risk, as these conditions promote fungal overgrowth and increase susceptibility to infections ranging from superficial mucosal disease to disseminated hematogenous candidiasis [22]. Healthcare-associated *Candida* sp. infections pose a continuing clinical challenge due to delayed diagnosis and poor treatment responsiveness [23]. Therefore, rapid and reliable detection of *C. albicans* is therefore essential to support timely preventive and therapeutic interventions in patients [24].

Routine identification still relies heavily on phenotypic techniques, including culture-based assays and microscopy, microscopic observation of colony morphology, and biochemical assays for species identification [25]. More advanced molecular approaches, such as polymerase chain reaction (PCR), real-time PCR, and immunoassays, have also been applied for detection [26]. Despite their accuracy, these methods are hindered by complex protocols, long turnaround times, reliance on specialized expertise, multi-step laboratory workflows, and high equipment costs [27,28].

In addition to diagnostic studies, previous research has examined materials influencing *C. albicans* colonization. For example, polymethyl methacrylate (PMMA), commonly used in dentistry, has been investigated in combination with oleic acid as a biofilm-repellent surface [29]. PMMA is a widely used denture base material and provides a favorable substrate for *C. albicans* attachment and biofilm formation [30]. In denture wearers, the inner surface of PMMA-based prostheses is highly susceptible to fungal colonization [31]. The hydrophobicity and relative surface roughness of PMMA further facilitate *C. albicans* adhesion and accumulation [32]. These findings emphasize the dual challenge posed by *C. albicans* in both clinical infections and biomaterial-associated colonization, highlighting the need for innovative and rapid diagnostic platforms to enhance early detection. To address the limitations of current diagnostic methods, this research proposes the development of a biosensor based on molecularly imprinted polymer (MIP) technology for the detection of *C. albicans*. In this approach, polymers are synthesized using functional monomers, a crosslinker, an initiator, and a suitable solvent in optimized ratios [33,34]. Methyl methacrylate (MMA) is incorporated as one of the main components to provide stability and functional compatibility with the target [35]. The polymer is imprinted with *C. albicans* templates directly on the electrode surface, followed by a self-assembly process that generates highly selective binding sites for the target organism [36]. Compared to conventional techniques, this method offers significant advantages, including simple electrode preparation, lower material and instrumentation costs, and ease of analysis [37,38]. Experimental results demonstrated a calculated limit of detection (LOD) of 1.30 CFU/mL, with an analytical range spanning 1–10,000 CFU/mL in buffer, using cyclic voltammetry as the electrochemical detection method.

## 2. Materials and Methods

### 2.1. Preparation of C. albicans Template

In this study, *C. albicans* was prepared by first isolating a single colony from the central stock culture. The colony was inoculated into 10 mL of tryptic soy broth (TSB) (105459, Merck, Darmstadt, Germany) and incubated overnight at 37 °C to allow for sufficient growth and increase in cell concentration. The resulting bacterial suspension was then quantified using two complementary approaches: optical density (OD600, Implen GmbH, Munich, Germany) measurement and colony-forming units (CFU) enumeration via the spread plate technique. Based on these analyses, the stock concentration was approximately 2.8 × 10^7^ CFU/mL. To preserve the cellular morphology for imprinting, the cells of *C. albicans* were fixed using 2.5% glutaraldehyde (354400, Sigma-Aldrich, St. Louis, MO, USA) for 2.5 h. A graded ethanol dehydration series was performed by immersing the cells in 50%, 60%, 70%, 80%, and 90% ethanol for 15 min each, followed by two treatments with 100% ethanol for 30 min. Residual ethanol (459836, Sigma-Aldrich, St. Louis, MO, USA) was removed by washing the samples twice with phosphate-buffered saline (PBS), and the final cell suspension was prepared in PBS for further use.

### 2.2. Preparation of Polymer Synthesis for Detection

For polymer synthesis, graphene oxide (GO) was incorporated at 0.15 mg/mL in a 2:3 ratio with the polymer matrix to enhance electrical conductivity [39]. The polymerization conditions were optimized by varying the ratios of the functional monomers methyl methacrylate (MMA) (M55909, Merck, Darmstadt, Germany) and acrylamide (AAM) (A8887, Sigma-Aldrich, St. Louis, MO, USA), as summarized in Table 1. To reinforce the structural integrity of the polymer, 45 mg of DHEBA (N,N′-(1,2-dihydroxyethylene)bis(acrylamide)) (294381, Sigma-Aldrich, St. Louis, MO, USA) was used as the cross-linking. Meanwhile, 1.5 mg AIBN (azobis(isobutyronitrile)) (768375, Sigma-Aldrich, St. Louis, MO, USA) served as the radical initiator. All components were dissolved in 300 μL DMSO (dimethyl sulfoxide) (W387509, Sigma-Aldrich, St. Louis, MO, USA) providing a homogeneous reaction environment. The prepared mixture provided a uniform and conductive polymer matrix suitable for imprinting and subsequent sensor fabrication. By systematically adjusting the monomer ratios while maintaining consistent concentrations of cross-linker, initiator, and solvent, the synthesis conditions were tailored to achieve optimal polymer properties. This approach aimed to balance selectivity, mechanical stability, and electrochemical performance of the final molecularly imprinted polymer (MIP)-based sensor designed for *C. albicans* detection.

After mixing, the polymerization reaction was performed by heating the reaction mixture to approximately 70 °C for 15 min., allowing the system to reach the gel point and form a stable pre-polymer structure suitable for subsequent imprinting. Achieving this stage was essential to ensure the stability of the polymer matrix, which was subsequently used in the following steps of sensor fabrication.

### 2.3. Polymer Imprinting on Screen Printed Electrode

**Figure 1:**
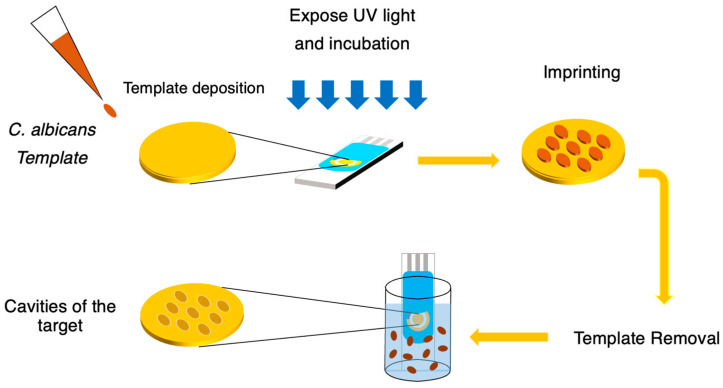
Schematic illustration of the preparation procedure of the screen-printed electrode for *C. albicans* detection. Where brown color represents the template imprinting, while light brown color corresponds to the cavities formed in the template after the imprinting process.

Screen-printed electrodes (SPEs) (DRP-220BT, Dropsens, Asturias, Spain) were employed for sensor fabrication. Each SPE consisted of a 4 mm diameter gold working electrode (WE), a gold counter electrode (CE), and a silver reference electrode (RE). Prior to the imprinting process, the pre-polymer gel mixed with graphene oxide (GO), as described in Section 2.2, was prepared. A 1 µL aliquot of the pre-polymer/GO mixture was carefully deposited onto the gold WE surface followed by 1 µL of fixed *C. albicans* cells in Section 2.1, was placed on top of the pre-polymer–GO layer. The electrode was then exposed to UVA (400 nm) for 3 h to initiate polymerization and promote template incorporation. After UV treatment, the electrode was incubated in a 60 °C oven for 18 h to complete the self-assembly process [40]. Template removal was carried out by washing the electrodes with 10% acetic acid at 30 °C for 30 min, followed by a final wash with Milli-Q water for 30 min, generating selective recognition cavities complementary to *C. albicans* as shown in schematic Figure 1.

### 2.4. Electrochemical Sensors Characterization

Electrochemical measurements were conducted using a µStat 400 bipotentiostat meter (Methrom, Dropsens, Asturias, Spain) to evaluate the detection of *C. albicans* at different concentration levels through cyclic voltammetry (CV). *C. albicans* suspensions were diluted in phosphate-buffered saline (PBS) to concentrations ranging from 1 to 10^4^ CFU/mL. A redox couple consisting of 1:1 K_4_[Fe(CN)_6_] and K_3_[Fe(CN)_6_], was used as electroactive probe. Cyclic voltammetry (CV) was performed within a potential window from −0.5 V to +0.7 V at a scan rate of 50 mV/s. The resulting current responses were analyzed to evaluate the analytical performance and detection capability of the fabricated MIP-based electrochemical sensor. The response time of the developed sensor was evaluated by measuring the time required for the electrochemical signal to reach a stable current response after the introduction of *C. albicans* at different concentration levels. The sensor exhibited a rapid response time of approximately 2 min, indicating its suitability for rapid bacterial detection. The limit of detection (LOD) was calculated according to the equation

$$LOD=\frac{3\sigma }{S}$$

where σ represents the standard deviation of the blank signal and S is the slope of the calibration curve. The LOD of the developed sensor was determined to be 1.30 CFU/mL.

## 3. Results

### 3.1. C. albicans Imprint on Gold SPE

**Figure 2:**
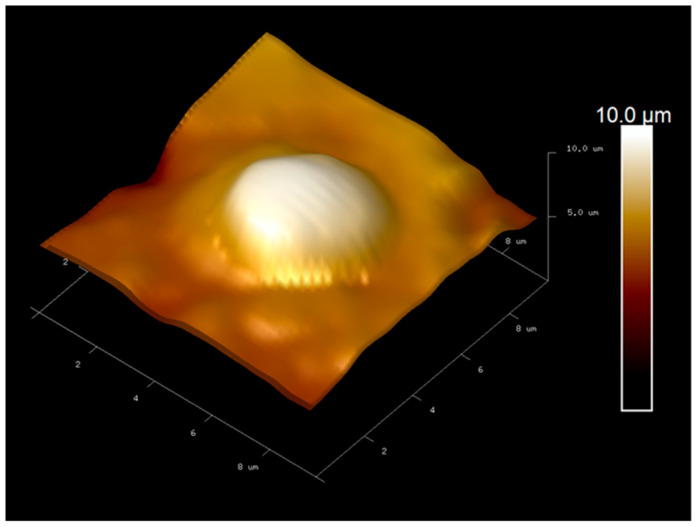
AFM image of the gold SPE surface after polymerization, illustrating *C. albicans* cells embedded within the MIP layer.

**Figure 3:**
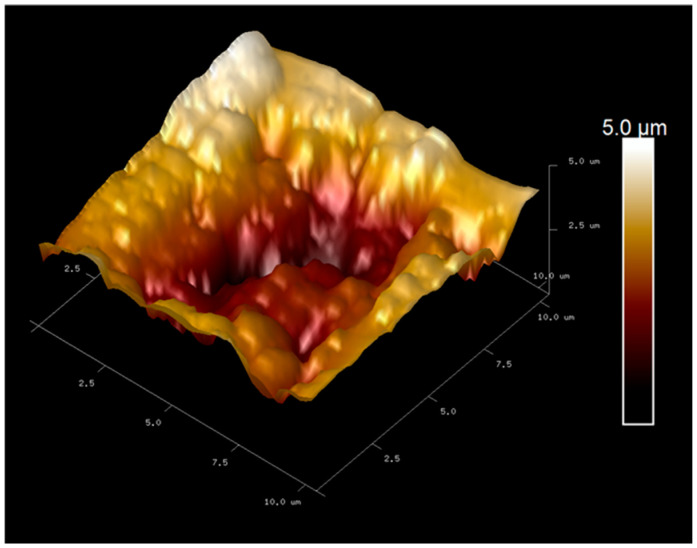
AFM image of the gold SPE surface after removal of the *C. albicans* template, revealing well-defined imprint cavities (~5.5 µm) corresponding to the fungal morphology.

Atomic force microscopy (AFM) was employed to investigate the surface morphology of the screen-printed electrode (SPE) after the imprinting process, with the purpose of verifying the successful formation of *C. albicans* recognition cavities within the polymer layer. As shown in Figure 2, intact *C. albicans* cells embedded on the gold SPE surface after polymerization confirming their proper template incorporation as a template. After template removal, the AFM image in Figure 3 shows well-defined cavities corresponding to the imprinted fungal cells. The cavities diameter was approximately 5.5 µm in diameter, consisting with the typical size of *C. albicans*. These results confirm that the imprinting procedure generated specific recognition sites and reproducible recognition sites suitable for selective detection of *C. albicans.*

### 3.2. Cyclic Voltammetry of C. albicans Detection

**Figure 4:**
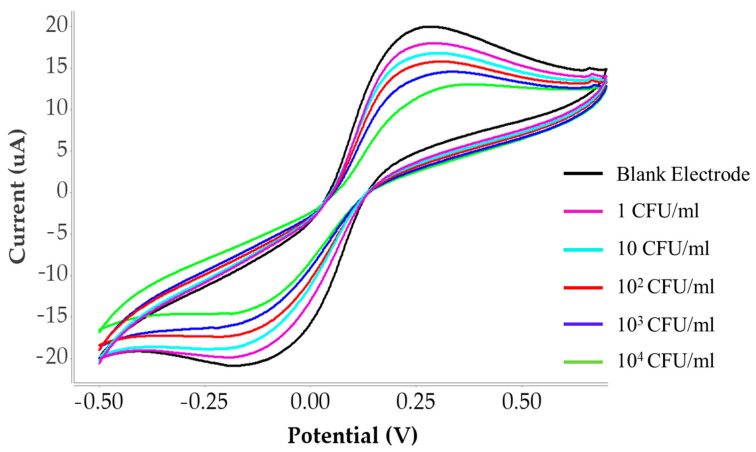
Cyclic voltammograms of *C. albicans* detection (1–10^4^ CFU/mL) using the optimized polymer synthesis condition (condition 4) at a scan rate of 50 mV/s from –0.5 to +0.7 V.

**Figure 5:**
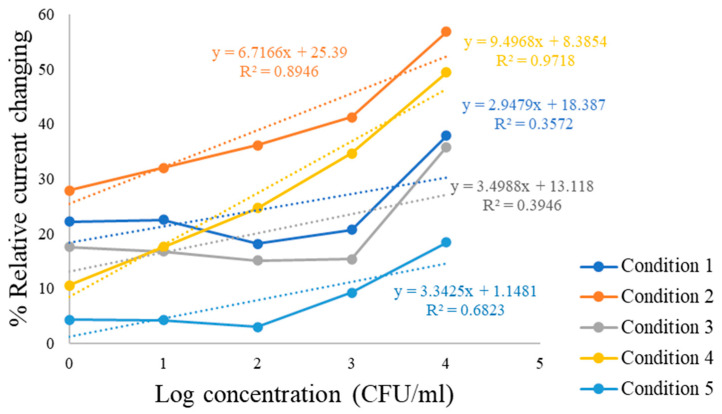
Calibration curves showing the linear relationship between log concentration of *C. albicans* (1–10^4^ CFU/mL) and percent current change under five polymer synthesis conditions, where the dotted line represents the linear curve for each condition.

Cyclic voltammetry (CV) was performed to evaluate the sensor response toward different concentration of *C. albicans* (1–10^4^ CFU/mL) diluted in PBS solution, with a blank solution serving as a reference. The resulting cyclic voltammograms (Figure 4 show that the blank sample (black curve) exhibited the highest current response, while increasing concentrations of *C. albicans* produced a gradual decrease in peak current. This decrease reflects hindered electron transfer due to increased surface coverage of fungal cells. This trend is illustrated by the sequential shifts in the CV curves, represented in purple, light blue, red, blue, and green, corresponding to increasing fungal concentrations. The reduction in current indicates that higher cell concentrations hinder electron transfer at the electrode surface. Quantitative current response data across the different concentrations and under five polymer synthesis conditions are summarized in Table 2, confirming the sensitivity of the developed sensor. The linear calibration curves for each polymer composition are shown in Figure 5. The relationship between the logarithmic concentration of *C. albicans* (CFU/mL) on the x-axis and the corresponding current response (µA) on the y-axis. Linear calibration curves were obtained for all five polymer synthesis conditions, demonstrating measurable sensor responses. Conditions 1, 3, and 5 (MMA:AAM ratios of 1:2, 2:3, and 1:1, respectively), yielded only modest change in current, particularly at concentrations above 10^3^ CFU/mL and the relatively low correlation coefficients observed in some conditions suggest that the imprinting process was not sufficiently optimized, resulting in weaker binding interactions between the polymer matrix and the target microorganism. In contrast, conditions 2 and 4 (MMA:AAM ratios of 2:1 and 3:2) exhibited stronger analytical responses across the entire concentration range. Among these, condition 4 provided the highest sensitivity, with a calibration slope of 9.4968 and R^2^ value of 0.9718, indicating superior linearity and detection capability compared to condition 2 (slope 6.7166; R^2^ = 0.8946).

### 3.3. Selectivity Test of C. albicans Detection

**Figure 6:**
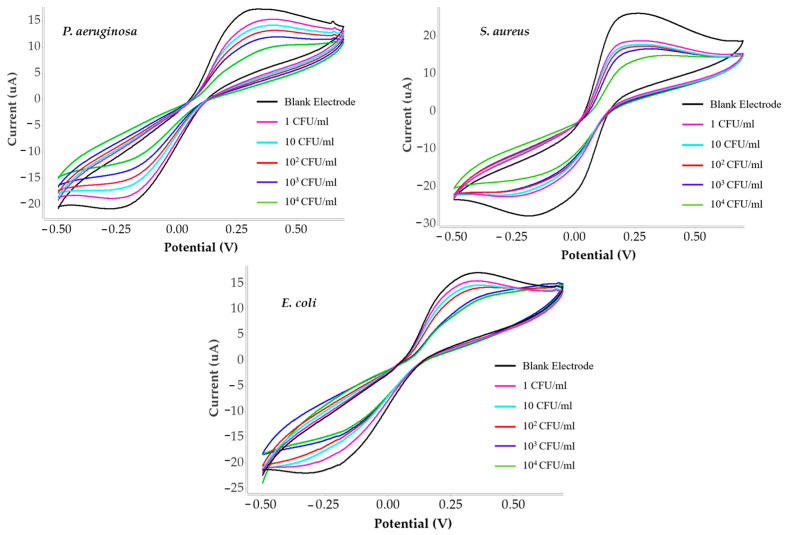
Cyclic voltammogram comparison showing sensor responses toward non-target bacteria (*E. coli*, *P. aeruginosa*, and *S. aureus*) for selectivity evaluation.

Selectivity was evaluated by testing the sensor against *Staphylococcus aureus* (Gram-positive), Pseudomonas aeruginosa and *Escherichia coli* (Gram-negative) under the same conditions used for *C. albicans*. CV responses are shown in Figure 6, and corresponding current data are provided in Table 3. In addition, two Gram-negative bacteria, were chosen as negative controls due to their distinct morphological sizes and shapes compared to *C. albicans.* Increasing concentrations of each bacterial strain were tested in separate experiments to assess cross-reactivity and to compare their sensor responses with those obtained for *C. albicans*.

**Figure 7:**
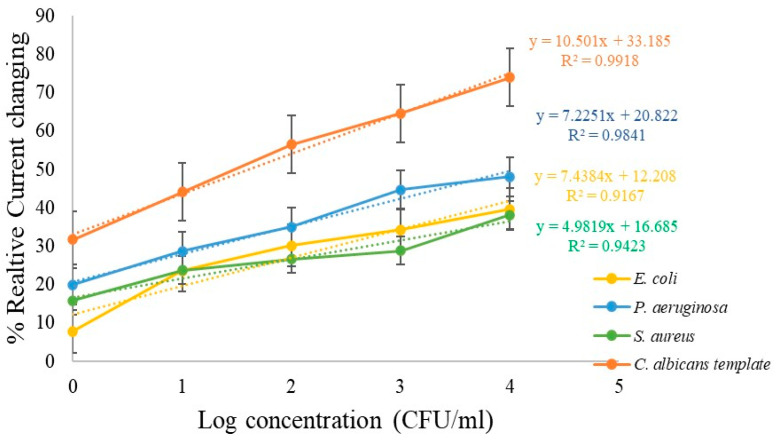
Percent current change as a function of log concentration for *C. albicans* (condition 4), compared with negative-control bacteria (*E. coli*, *P. aeruginosa*, and *S. aureus*).

Furthermore, Figure 7 shows the linearity ranges of the calibration curves obtained for each microorganism. The results confirm that the sensor exhibited a greater current suppression in the presence of *C. albicans* compared to *S. aureus*, *P. aeruginosa*, and *E. coli*, thereby demonstrating the high selectivity of the molecularly imprinted polymer (MIP)-based sensor.

The linearity ranges of percent current change for the negative control bacteria *Escherichia coli* (*E. coli*), *Pseudomonas aeruginosa* (*P. aeruginosa*), and *Staphylococcus aureus* (*S. aureus*) demonstrated consistently low responses across all tested concentrations when compared with *C. albicans* within the range of 1–10^4^ CFU/mL. This clear difference highlights the ability of the developed sensor to specifically recognize *C. albicans* over other microbial species.

The sensitivity of the sensor was determined from the slope of the calibration curve obtained by plotting the relative current change (%) versus the logarithmic concentration of *C. albicans*. The sensitivity was found to be the highest at 10.50% log^−1^(CFU/mL) for *C. albicans* detection. In contrast, the sensitivities for the negative controls were markedly lower, with values of 7.44% log^−1^(CFU/mL) for *E. coli*, 7.23% log^−1^(CFU/mL) for *P. aeruginosa*, and 4.98% log^−1^(CFU/mL) for *S. aureus.* These findings confirm that the molecularly imprinted polymer (MIP)-based sensor, when integrated with a gold screen-printed electrode (SPE), exhibits superior sensitivity and selectivity toward *C. albicans* compared to other tested microorganisms. The results further validate the effectiveness of the imprinting strategy in producing recognition sites that closely match the size and morphology of *C. albicans*, thereby minimizing interference from non-target bacteria.

### 3.4. Comparison of NIP and MIP Sensor

**Figure 8:**
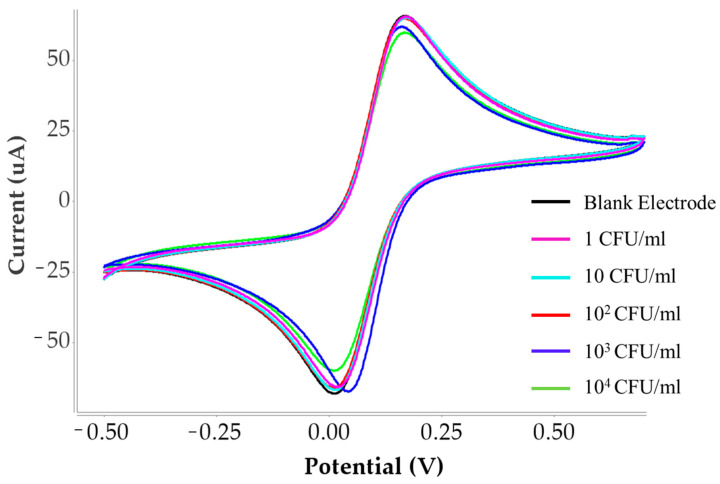
Cyclic voltammograms of the non-imprinted polymer (NIP)-modified electrode in the presence of *C. albicans*.

**Figure 9:**
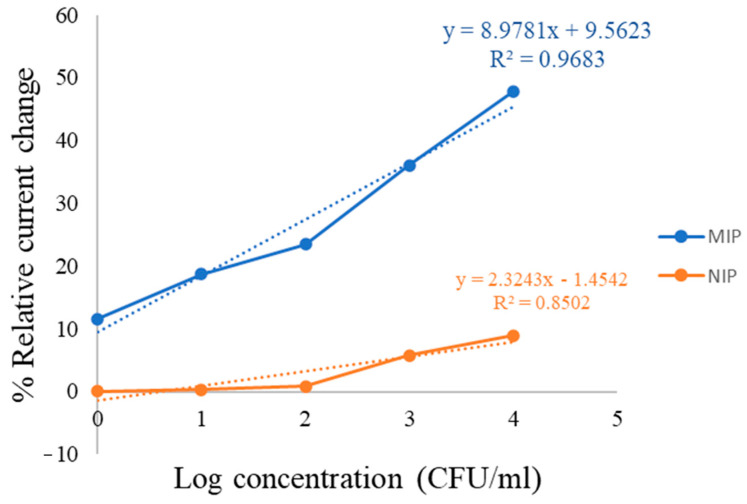
Comparison of the linear calibration curves of MIP and NIP sensors for the detection of *C. albicans*.

To verify the effectiveness of the imprinting process, the analytical performance of the MIP sensor was compared with a non-imprinted polymer (NIP) sensor prepared under same experimental condition with condition 4. The corresponding cyclic voltammetry curves are shown in Figure 8 and the linear comparison is shown in Figure 9.

The MIP sensor exhibited a significantly higher response toward *C. albicans* compared with the NIP sensor. This difference can be attributed to the presence of specific recognition cavities in the MIP structure that were formed during the imprinting process and are complementary in shape and chemical functionality to the *C. albicans* detection. In contrast, the NIP sensor lacks these specific binding sites and therefore mainly exhibits non-specific adsorption, resulting in a much weaker signal response. The calibration curves further demonstrate this difference, where the slope obtained for the MIP sensor is significantly higher than that of the NIP sensor (8.9781) is significantly higher than that of the NIP sensor (2.3243). A larger slope indicates higher sensitivity of the sensing platform toward *C. albicans*. The enhanced sensitivity of the MIP sensor suggests that the imprinting process successfully created selective binding sites that facilitate the effective recognition and capture of the target microorganism.

These results clearly demonstrate that the imprinting strategy plays a crucial role in improving the sensing performance. The significantly higher response and steeper calibration slope observed for the MIP sensor confirm the successful formation of specific recognition cavities and highlight the advantage of the MIP-based sensor over the non-imprinted control.

### 3.5. Reproducibility of the Sensor

**Figure 10:**
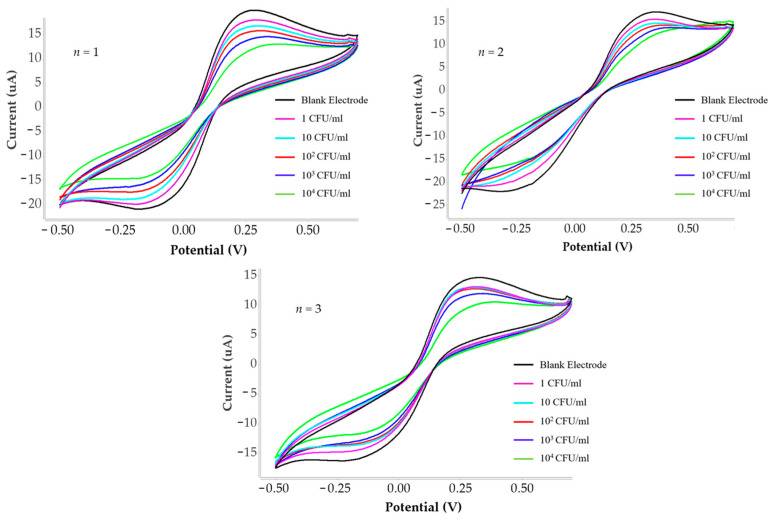
Cyclic voltammograms obtained from three repeated measurements for *C. albicans* detection under the same polymer synthesis conditions (Condition 4).

The reproducibility of the proposed sensor was evaluated to assess the reliability of the fabrication process and the consistency of the sensing performance. For this purpose, several independently prepared MIP-modified electrodes (*n* = 3) were fabricated under identical experimental conditions and used to detect *C. albicans* at the same concentration. As shown in Figure 10 and current data responses shown in the Table 4, the obtained responses from different electrodes were highly consistent, indicating good reproducibility of the sensor fabrication process.

The relative standard deviation (RSD) of the measured signals was calculated to further evaluate the reproducibility as shown in the Table 5. The results showed a low RSD value of approximately 3.47% to −8.83% across the concentration range of 1–10,000 CFU/mL, demonstrating that the proposed sensing platform exhibits excellent reproducibility. This good reproducibility can be attributed to the stable structure of the molecularly imprinted polymer and the consistent preparation procedure of the sensing layer. These results confirm that the developed MIP-based sensor can provide reliable and repeatable detection of *C. albicans*.

### 3.6. Comparison with Previously Reported Methods for C. albicans Detection

To evaluate the analytical performance of the proposed sensor, the detection capability of the developed MIP-based electrochemical sensor was compared with previously reported methods for *C. albicans* detection, as summarized in Table 6. The proposed sensor demonstrated a low limit of detection (LOD) of 1.30 CFU/mL with linear detection range of 1–10^4^ CFU/mL, indicating high sensitivity for the target. Compared with previously reported electrochemical biosensors, such as the MIP-based electrochemical sensor with a detection limit of 2.8 CFU/mL and a linear range of 10^4^–10^7^ CFU/mL [41], the proposed sensor exhibits improved sensitivity, particularly in the lower concentration range. Similarly, an electrochemical immunosensor based on electrochemical impedance spectroscopy (EIS) reported a detection limit of 10 CFU/mL with a linear range of 10^1^–10^8^ CFU/mL [42], which is higher than that obtained in this study. Molecular detection techniques such as real-time PCR (qPCR) and PCR–lateral flow assays have also been widely used for the detection of *C. albicans*. These methods typically provide high analytical sensitivity, with reported detection limits of approximately 10 CFU/mL and 2 CFU/mL, respectively [43,44]. However, PCR-based techniques generally require complex instrumentation, longer analysis time, and skilled personnel.

Overall, the proposed MIP-based electrochemical sensor demonstrates competitive analytical performance compared with previously reported methods. In addition to its high sensitivity, the developed platform offers advantages such as operational simplicity, rapid detection, and the absence of biological recognition elements, highlighting its potential for practical applications in the rapid detection of *C. albicans*.

## 4. Discussion

The evaluation of polymer synthesis under five different monomer ratios showed that condition 4, consisting of methyl methacrylate (MMA) and acrylamide (AAM) at a 3:2 ratio, yielded the most effective performance for *C. albicans* detection in PBS. This result is consistent with chemical characteristics of MMA, which polymerizes into polymethyl methacrylate (PMMA). PMMA contains methacrylic acid derived caboxyl groups and methanol derived hydroxyl functionalities that contribute to its mechanical strength, structural rigidity and stability. These properties support the formation of a robust polymer matrix suitable for imprinting intact *C. albicans* cells. Atomic force microscopy (AFM) used for confirmed the successful imprinting of fungal templates on the screen printed electrode images revealed well-defined cavities on the screen-printed electrode surface. After template removal, well-defined cavities consistent with the morphology of *C. albicans* were observed, indicating that the polymerization and imprinting steps generated specific recognition sites capable of selectivity capturing fungal cells during sensing. Cyclic voltammetry measurements further validated the imprinting process. The progressive decrease in current with increasing *C. albicans* concentration reflected restricted electron transfer resulting from target binding to the imprinted cavities. as distinct current responses were observed across the tested concentration range. Notably, the current decreased progressively with increasing *C. albicans* concentrations in PBS, reflecting successful binding of the target organism to the imprinted electrode surface. This result highlights the sensitivity of the optimized polymer matrix in distinguishing varying levels of *C. albicans.* The selectivity of the sensor was assessed by comparing responses to *C. albicans* with those obtained from *E. coli*, *P. aeruginosa*, and *S. aureus*. Across all tested concentrations, the percent current changes for these non-target bacteria were significantly lower than those observed for *C. albicans*. This clear contrast highlights the specificity of the molecularly imprinted polymer (MIP)-based sensor and confirms that the recognition sites preferentially interact with the target fungal cells rather than structurally dissimilar bacteria species. Overall, the findings indicate that the polymer formulation used in condition 4 provides an effective balance of mechanical stability, imprinting fidelity, sensitivity, and selectivity These characteristics make it a promising material for the reliability electrochemical detection of *C. albicans* and support its suitability for development into a rapid and low cost diagnostic platform.

## 5. Conclusions

This study demonstrates a rapid and effective approach for the detection of *C. albicans* using a molecularly imprinted polymer (MIP)-based imprinting strategy. By optimizing the polymer formulation, the sensor was tailored to enhance analytical performance for potential on-site screening in hospital settings. Among the tested compositions, the polymer prepared with methyl methacrylate (MMA) and acrylamide (AAM) at a 3:2 ratio exhibited the most favorable analytical behavior toward *C. albicans*. The sensor achieved high sensitivity, showing a current change of 10.50% per logarithmic concentration increment, alongside strong linearity with a correlation coefficient (R2) of 0.9918. Furthermore, the calculated limit of detection of 1.30 CFU/mL confirmed its capability to detect very low fungal concentrations. Selectivity assessment further demonstrated that the sensor produced markedly higher current responses for *C. albicans* than for the control bacteria, underscoring the specificity of the imprinting process. Although the present evaluation was conducted in phosphate-buffered saline (PBS), the promising results highlight the method’s potential for further clinical application.

Overall, this work provides a practical, sensitive, and rapid detection strategy for *C. albicans*, supporting the potential of MIP-based electrochemical biosensors as valuable tools for early diagnosis and infection management in healthcare environments.

## Figures and Tables

**Table 1:** Monomer compositions and polymer synthesis conditions used for fabricating the molecularly imprinted polymer (MIP).

Condition	Ratio (n:n)	MMA (µL)	AAM (mg)
1	1:2	10.7	14.2
2	2:1	21.4	7.1
3	2:3	21.4	21.3
4	3:2	32.1	14.2
5	1:1	10.7	7.1

**Table 2:** Current response values obtained from cyclic voltammetry measurements of *C. albicans* across five polymer synthesis conditions.

**Condition 1**	**Current (µA)**	**∆I (µA)**	**% Current Changing**
Blank	41.13		
1 CFU/mL	31.99	9.14	22.21
10 CFU/mL	31.86	9.27	22.53
100 CFU/mL	33.68	7.45	18.12
1000 CFU/mL	32.62	8.50	20.67
10,000 CFU/mL	25.55	15.58	37.88
**Condition 2**	**Current (µA)**	**∆I (µA)**	**% Current Changing**
Blank	24.30		
1 CFU/mL	17.52	6.78	27.91
10 CFU/mL	16.52	7.77	31.99
100 CFU/mL	15.53	8.77	36.09
1000 CFU/mL	14.27	10.03	41.27
10,000 CFU/mL	10.48	13.81	56.85
**Condition 3**	**Current (µA)**	**∆I (µA)**	**% Current Changing**
Blank	38.89		
1 CFU/mL	32.05	6.84	17.59
10 CFU/mL	32.38	6.51	16.74
100 CFU/mL	33.02	5.87	15.10
1000 CFU/mL	32.91	5.98	15.38
10,000 CFU/mL	24.98	13.91	35.76
**Condition 4**	**Current (µA)**	**∆I (µA)**	**% Current Changing**
Blank	17.45		
1 CFU/mL	15.61	1.84	10.52
10 CFU/mL	14.38	3.07	17.57
100 CFU/mL	13.14	4.31	24.67
1000 CFU/mL	11.40	6.05	34.68
10,000 CFU/mL	8.82	8.63	49.45
**Condition 5**	**Current (µA)**	**∆I (µA)**	**% Current Changing**
Blank	54.37		
1 CFU/mL	52.05	2.31	4.26
10 CFU/mL	52.08	2.28	4.20
100 CFU/mL	52.73	1.63	3.00
1000 CFU/mL	49.32	5.04	9.27
10,000 CFU/mL	44.35	10.02	18.43

**Table 3:** Current response values of non-target bacteria (*E. coli*, *P. aeruginosa*, *S. aureus*) for selectivity comparison with *C. albicans*.

***E. coli***	**Current (µA)**	**∆I (µA)**	**% Relative Current Change**
Blank	17.86		
1 CFU/mL	16.47	1.39	7.77
10 CFU/mL	13.64	4.22	23.63
100 CFU/mL	12.48	5.38	30.11
1000 CFU/mL	11.74	6.12	34.28
10,000 CFU/mL	10.78	7.08	39.64
***P. aeruginosa***	**Current (µA)**	**∆I (µA)**	**% Relative Current Change**
Blank	13.33		
1 CFU/mL	10.67	2.66	19.98
10 CFU/mL	9.51	3.82	28.65
100 CFU/mL	8.67	4.66	34.98
1000 CFU/mL	7.38	5.95	44.65
10,000 CFU/mL	4.92	8.41	63.11
***S. aureus***	**Current (µA)**	**∆I (µA)**	**% Relative Current Change**
Blank	47.45		
1 CFU/mL	39.94	7.51	15.82
10 CFU/mL	36.18	11.27	23.75
100 CFU/mL	34.82	12.64	26.63
1000 CFU/mL	33.75	13.70	28.86
10,000 CFU/mL	29.34	18.11	38.17

**Table 4:** Current response values of three repeated measurements for *C. albicans* detection under the same polymer synthesis conditions.

**Reproducibility 1**	**Current (µA)**	**∆I (µA)**	**% Current Changing**
Blank	17.45		
1 CFU/mL	15.61	1.84	10.52
10 CFU/mL	14.38	3.07	17.57
100 CFU/mL	13.14	4.31	24.67
1000 CFU/mL	11.40	6.05	34.68
10,000 CFU/mL	8.82	8.63	49.45
**Reproducibility 2**	**Current (µA)**	**∆I (µA)**	**% Current Changing**
Blank	17.68		
1 CFU/mL	15.46	2.22	12.56
10 CFU/mL	14.18	3.50	19.80
100 CFU/mL	13.77	3.91	22.14
1000 CFU/mL	11.07	6.61	37.39
10,000 CFU/mL	9.53	8.16	46.13
**Reproducibility 3**	**Current (µA)**	**∆I (µA)**	**% Current Changing**
Blank	17.57		
1 CFU/mL	15.54	2.03	11.55
10 CFU/mL	14.28	3.29	18.73
100 CFU/mL	13.45	4.12	23.45
1000 CFU/mL	11.23	6.34	36.08
10,000 CFU/mL	9.17	8.40	47.81

**Table 5:** Average current response and relative standard deviation (RSD) of the MIP-based electrochemical biosensor at different concentrations of *C. albicans* (n = 3).

Concentration	Average (µA)	SD	% RSD
1 CFU/mL	11.54	1.02	8.83
10 CFU/mL	18.70	1.11	5.96
100 CFU/mL	23.42	1.27	5.42
1000 CFU/mL	36.05	1.36	3.76
10,000 CFU/mL	47.79	1.66	3.47

**Table 6:** Comparison of the analytical performance of previously reported methods for *C. albicans* detection.

Method	LOD	Linear Range	Reference
This work	1.30 CFU/mL	1–10^4^	This work
Electrochemical MIP biosensor	2.8 CFU/mL	10^4^–10^7^ CFU/mL	[41]
Electrochemical immunosensor (EIS)	10 CFU/mL	10^1^–10^8^ CFU/mL	[42]
Real-time PCR (qPCR)	10 CFU/mL	–	[43]
PCR-Lateral flow assay	2 CFU/mL	–	[44]

## Data Availability

The original contributions presented in this study are included in the article. Further inquiries can be directed to the corresponding author.

## Abbreviations

The following abbreviations are used in this manuscript:

AAM	Acrylamide
AIBN	Azobisisobutyronitrile
DMSO	Dimethyl Sulfoxide
DJD	Degenerative Joint Diseases
EGDMA	Ethylene Glycol Dimethacrylate
ELISA	Enzyme-linked immunosorbent assays
GO	Graphene Oxide
LOD	Limit of Detection
LOQ	Limit of Quantity
MIP	Molecularly Imprinted Polymer
OA	Osteoarthritis
PCR	polymerase chain reaction
RA	Rheumatoid arthritis
SPE	Screen-Printed Electrode
TMJ	temporomandibular joint
UPLC-MS/MS	Ultraperformance liquid chromatography–tandem mass spectrometry
UV	Ultraviolet
VP	N-vinylpyrrolidone

## References

[1] Malavika G., Ravi S.S.S., Maheswary D., Leela K.V., Lathakumari R.H., Lekshmi P.K. (2025). Role of *Candida albicans* in chronic inflammation and the development of oral squamous cell carcinoma. Cancer Pathog. Ther..

[2] Macias-Paz I.U., Pérez-Hernández S., Tavera-Tapia A., Luna-Arias J.P., Guerra-Cárdenas J.E., Reyna-Beltrán E. (2023). *Candida albicans* the main opportunistic pathogenic fungus in humans. Rev. Argent. Microbiol..

[3] Sahoo B., Goyal R., Dutta S., Joshi P., Sanyal K. (2025). *Candida albicans*: Insights into the Biology and Experimental Innovations of a Commonly Isolated Human Fungal Pathogen. ACS Infect. Dis..

[4] Gaffar N.R., Valand N., Venkatraman Girija U. (2025). Candidiasis: Insights into Virulence Factors, Complement Evasion and Antifungal Drug Resistance. Microorganisms.

[5] Neal C.M., Gonzalez A.I., Oshinowo A., Fazle T., Kako T. (2025). Recurrent vulvovaginal candidiasis associated with the use of a probiotic supplement. IDCases.

[6] Satora M., Grunwald A., Zaremba B., Frankowska K., Żak K., Tarkowski R., Kułak K. (2023). Treatment of Vulvovaginal Candidiasis-An Overview of Guidelines and the Latest Treatment Methods. J. Clin. Med..

[7] Benarrós M.S., Salvarani F.M. (2024). Candidiasis in *Choloepus* sp.—A Review of New Advances on the Disease. Animals.

[8] Kashyap B., Padala S.R., Kaur G., Kullaa A. (2024). *Candida albicans* Induces Oral Microbial Dysbiosis and Promotes Oral Diseases. Microorganisms.

[9] Mouratidou C., Tsakiri K., Dourliou V., Marneri A., Stougianni M., Pavlidis E. (2025). Early-Onset Candidemia in Adult Intensive Care Units. Diagnostics.

[10] Katsipoulaki M., Stappers Mark H.T., Malavia-Jones D., Brunke S., Hube B., Gow Neil A.R. (2024). *Candida albicans* and *Candida glabrata*: Global priority pathogens. Microbiol. Mol. Biol. Rev..

[11] Srb N., Talapko J., Meštrović T., Fureš R., Stupnišek M., Srb A.M., Škrlec I. (2025). A Comprehensive Overview of *Candida albicans* as the Leading Pathogen in Vulvovaginal Candidiasis. J. Fungi.

[12] Czechowicz P., Nowicka J., Gościniak G. (2022). Virulence Factors of *Candida* spp. and Host Immune Response Important in the Pathogenesis of Vulvovaginal Candidiasis. Int. J. Mol. Sci..

[13] Lashkarbolouk N., Mazandarani M., Hojjati M., Hosseini S.S. (2025). Investigation of fungal nosocomial infections and antimicrobial drug resistance pattern after open-heart surgery; a cross-sectional study. Ann. Med. Surg..

[14] Khachab Y., Ghader M.B., Tahesh V., Khoumassi R., Sokhn E.S. (2026). Prevalent fungal pathogens and antifungal resistance in Lebanon: A scoping review. J. Infect. Public Health.

[15] Parambath S., Dao A., Kim H.Y., Zawahir S., Alastruey Izquierdo A., Tacconelli E., Govender N., Oladele R., Colombo A., Sorrell T. (2024). *Candida albicans*—A systematic review to inform the World Health Organization Fungal Priority Pathogens List. Med. Mycol..

[16] La Via L., Sangiorgio G., Stefani S., Marino A., Nunnari G., Cocuzza S., La Mantia I., Cacopardo B., Stracquadanio S., Spampinato S. (2024). The Global Burden of Sepsis and Septic Shock. Epidemiologia.

[17] Yu S.N., Hong S.I., Park J.W., Jeon M.H., Cho O.H. (2025). Epidemiology and Clinical Features of Candida Bloodstream Infections: A 10-Year Retrospective Study in a Korean Teaching Hospital. J. Fungi.

[18] Tan C., Wu A., Li C. (2025). Forty years of global research on WHO’s four critical priority fungal pathogens: Advances and prospects. J. Infect. Public Health.

[19] Wang L., Feng Y., Wang S., Shi L., Ren Y., Yang Z., Shi D. (2025). Successful management of recurrent cutaneous granulomas caused by *Candida albicans* using aminolevulinic acid photodynamic therapy post-surgery: A case report. Photodiagnosis Photodyn. Ther..

[20] Yang X., Jin X., Yang Z., Wang Y., Wei A., Yang X. (2024). Isolated Cutaneous Granuloma Caused by Candida Parapsilosis: Case Report and Literature Review. Mycopathologia.

[21] Strickland A.B., Shi M. (2021). Mechanisms of fungal dissemination. Cell. Mol. Life Sci..

[22] Garnacho-Montero J., Barrero-García I., León-Moya C. (2024). Fungal infections in immunocompromised critically ill patients. J. Intensive Med..

[23] Mallick D.C., Kaushik N., Goyal L., Mallick L., Singh P. (2025). A Comprehensive Review of Candidemia and Invasive Candidiasis in Adults: Focus on the Emerging Multidrug-Resistant Fungus Candida auris. Diseases.

[24] Chiang S.J.F., Chien M.K., Tsai C.Y., Hsiao J.C., Koo F.H., Yen Y.F., Chou Y.C., Cheng C. (2024). A Simple, Fast, and Reliable Method for the Identification of *Candida albicans*. Environ. Health Insights.

[25] Arbefeville S.S., Timbrook T., Garner C.D. (2024). Evolving strategies in microbe identification-a comprehensive review of biochemical, MALDI-TOF MS and molecular testing methods. J. Antimicrob. Chemother..

[26] Felix G.N., de Freitas V., da Silva Junior A., Magri M.M.C., Rossi F., Sejas O., Abdala E., Malbouisson L.M.S., Guimarães T., Benard G. (2023). Performance of a Real-Time PCR Assay for the Detection of Five Candida Species in Blood Samples from ICU Patients at Risk of Candidemia. J. Fungi.

[27] Cherie N., Berta D.M., Tamir M., Yiheyis Z., Angelo A.A., Mekuanint Tarekegn A., Chane E., Nigus M., Teketelew B.B. (2024). Improving laboratory turnaround times in clinical settings: A systematic review of the impact of lean methodology application. PLoS ONE.

[28] Edayan J.M., Gallemit A.J., Sacala N.E., Palmer X.-L., Potter L., Rarugal J., Velasco L.C. (2024). Integration technologies in laboratory information systems: A systematic review. Inform. Med. Unlocked.

[29] Petrović M., Randjelović M., Igić M., Randjelović M., Arsić Arsenijević V., Mionić Ebersold M., Otašević S., Milošević I. (2022). Poly(methyl methacrylate) with Oleic Acid as an Efficient *Candida albicans* Biofilm Repellent. Materials.

[30] Išljamović M., Bonvin D., Milojević M., Stojanović S., Spasić M., Stojković B., Janošević P., Otašević S., Ebersold M.M. (2024). Antifungal Effect of Poly(methyl methacrylate) with Farnesol and Undecylenic Acid against *Candida albicans* Biofilm Formation. Materials.

[31] Bohinc K., Zore A., Velikonja T., Rojko F., Štukelj R., Učakar A., Abram A., Matijaković Mlinarić N., Čekada M., Nikolić J. (2025). Antifungal Effect of Poly(methyl methacrylate) Coated with Polyelectrolyte Multilayers. ACS Omega.

[32] Al Hatem O., Ontiveros J.C., Belles D.M., Gonzalez M.D., van der Hoeven R. (2025). Surface Roughness and Microbial Adhesion on Four Provisional Prosthodontic Restorative Materials. Dent. J..

[33] Cutaia A., Alberti G. (2026). Green Synthesis of Molecularly Imprinted Polymers: Advances Toward Sustainable Materials. Polymers.

[34] Özsoylu D., Kholy E., Wagner P., Schöning M. (2026). Lipopolysaccharide-templated biomimetic molecularly imprinted polymer (MIP) surfaces for high-affinity bacterial capture. Chem. Eng. J..

[35] Sanchez Armengol E., Sánchez Soler L.A., Valverde Offermann N., Laffleur F. (2024). Polymer powerhouse: Methyl methacrylate–A breakthrough blend for superior adhesion to gingiva. Dent. Mater..

[36] Schaefer K.G., Russell C.M., Pyron R.J., Conley E.A., Barrera F.N., King G.M. (2024). Polymerization mechanism of the *Candida albicans* virulence factor candidalysin. J. Biol. Chem..

[37] Dong H., Wang Z., Chang A., Zhao M., Wang S., Li Y., Su C., Zhou J., Zhang Z., Zhou Y. (2025). A ratiometric electrochemical sensor based on molecular imprinting for highly selective detection of HVA. Microchem. J..

[38] Reidell A.C., Pazder K.E., LeBarron C.T., Stewart S.A., Hosseini S. (2024). Modified Working Electrodes for Organic Electrosynthesis. ACS Org. Inorg. Au.

[39] Vongmanee N., Nampeng J., Pintavirooj C., Visitsattapongse S. (2025). Biosensor Based on Electrochemical Analysis for *Staphylococcus aureus* Detection with Molecular Imprinted Polymer Technique. Polymers.

[40] Vongmanee N., Nampeng J., Rattanapithan K., Sriwichai P., Pintavirooj C., Visitsattapongse S. (2025). A Novel Approach for Optimizing Molecularly Imprinted Polymer Composition in Electrochemical Detection of Collagen Peptides. Bioengineering.

[41] Isbilir H., Kaya H.O., Tekintaş Y., Kurul F., Cetin A.E., Topkaya S.N. (2025). Enhanced electrochemical biosensing of *Candida albicans* via NiFe_2_O_4_ nanoparticle-doped imprinted polymers. Microchem. J..

[42] D’Aponte T., De Luca M., Sakač N., Schibeci M., Arciello A., Roscetto E., Catania M.R., Iannotti V., Velotta R., Della Ventura B. (2023). Rapid detection of *Candida albicans* in urine by an Electrochemical Impedance Spectroscopy (EIS)-based biosensor. Sens. Diagn..

[43] Busser F., Coelho V.C., Fonseca C.A., Del Negro G.M.B., Shikanai-Yasuda M.A., Lopes M.H., Magri M.M.C., Freitas V. (2020). A Real Time PCR strategy for the detection and quantification of *Candida albicans* in human blood. Rev. Do Inst. Med. Trop. São Paulo.

[44] Srichaiyapol O., Saiboonjan B., Ngernpimai S., Ponsue C., Sa-Ingthong N., Thongmee P., Wonglakorn L., Sukkasem C., Kendal R.P., Daduang J. (2025). Enhanced performances of the short-PCR coupled lateral flow assay in the detection of *Candida albicans* in clinical blood samples. Asian Pac. J. Allergy Immunol..

